# Preliminary study of sound touch elastography in diffuse thyroid disease in children

**DOI:** 10.3389/fped.2022.964413

**Published:** 2022-10-06

**Authors:** Lin Li, Aimei Zhang, Dan Chen, Benjamin H. Taragin, Xiaoyong Luo

**Affiliations:** ^1^Department of Ultrasonography, Suining Central Hospital, Suining, China; ^2^Department of Radiology, Samson Assuta Ashdod University Hospital, Ashdod, Israel

**Keywords:** children, ultrasound elastography, sound touch elastography, diffuse thyroid disease, Graves’ disease, Hashimoto’s thyroiditis

## Abstract

**Objective:**

The purpose of this study was to evaluate the use of sound touch elastography (STE) in conjunction with conventional ultrasound in the differential diagnosis of diffuse thyroid disease (DTD) and normal thyroid in children.

**Methods:**

Studies performed on 62 children with DTD and 30 normal volunteers were reviewed. Standard gray scale ultrasound, Doppler ultrasound and STE of the examinees, and the serum test results of children with DTD were collected, analyzed and compared.

**Results:**

The STE-Mean values in the Graves’ disease (GD) group, Hashimoto’s thyroiditis (HT) group, and normal control group, respectively, were 19.35 ± 5.00 kPa, 19.43 ± 6.06 kPa, and 11.24 ± 1.99 kPa. With an area under the ROC curve (AUC) of 0.945, STE-Mean values differentiated DTD from normal children. The peak systolic velocity (PSV) of the superior thyroid artery separated DTD from normal children and AUC from children with GD and HT, respectively, and was 0.992 and 0.864. The PSV of superior thyroid artery revealed a somewhat favorable connection with FT3 and FT4.

**Conclusion:**

The STE results revealed that thyroid stiffness was higher in children with DTD than in normal children, but further differentiation into GD and HT subgroups lacked specificity, and the superior thyroid artery flow velocity might be a good supplement to distinguish both.

## Introduction

The global incidence of autoimmune thyroid disease (AITD) is close to 5% (1, 2), often beginning in infancy, ultimately affecting children’s growth and learning. Diffuse thyroid disease (DTD) in children is mostly Graves’ disease (GD) and Hashimoto’s thyroiditis (HT), both of which are AITD. The diagnosis of DTD in children is mostly based on clinical symptoms, blood tests, and imaging investigations, with ultrasound as the initial imaging assessment, sometimes augmented by nuclear scan and CT. Ultrasonic examination has long been used for diagnosis and follow-up of diffuse and nodular thyroid diseases. However, GD and HT have overlapping ultrasound appearances (3, 4). For both GD and HT, gray-scale ultrasound can demonstrate increased thyroid volume and decreased echogenicity, while color Doppler demonstrates increased blood flow perfusion. The blood flow velocity of untreated GD is often higher than that of HT (5–7).

As a relatively new and developing method, ultrasonic elastography (UE) has developed into a reliable diagnostic tool in the last decade since it offers additional information on tissue stiffness to aid clinical diagnosis of various disorders. Many research studies have been performed in the adult population in various organ systems and locations. In recent years, there have been limited studies reported on children. Most of the published papers have focused on liver diseases including liver fibrosis, biliary atresia, liver congestion (8–10), A fair amount of projects described musculoskeletal, renal, pancreatic, lymph node, spleen, and neurologic imaging (11–17) has received less attention. DTD have rarely been discussed (11).

According to the European Federation of Societies of Ultrasound in Biomedicine (EFSUMB) Clinical Practice Guidelines and Recommendations for Elastography for Non-Liver Applications (2018) (18), UE is classified into two types: Strain elastography (SE) and shear wave elastography (SWE); SWE is further classified into transient elastography (TE), point shear wave elastography (pSWE), and multidimensional SWE (2D-SWE and 3D-SWE). The STE technique used in this study is 2D-SWE, in which the ultrasound probe generates shear waves by acoustic radiation force within the ROI sampling area, and continuously records the propagation velocity of those waves to calculate the elastic modulus of the tissues. The goal of our study was to see how STE paired with conventional gray scale and Doppler ultrasonography may help in the differential diagnosis of diffuse thyroid lesions in children.

## Materials and methods

### Patient and participant assessment

The Ethics Committee of Suining Central Hospital accepted this investigation, and all participants were younger than 18 years old, and their legal guardians consented to participate in this study and gave written informed consent.

**FIGURE 1:**
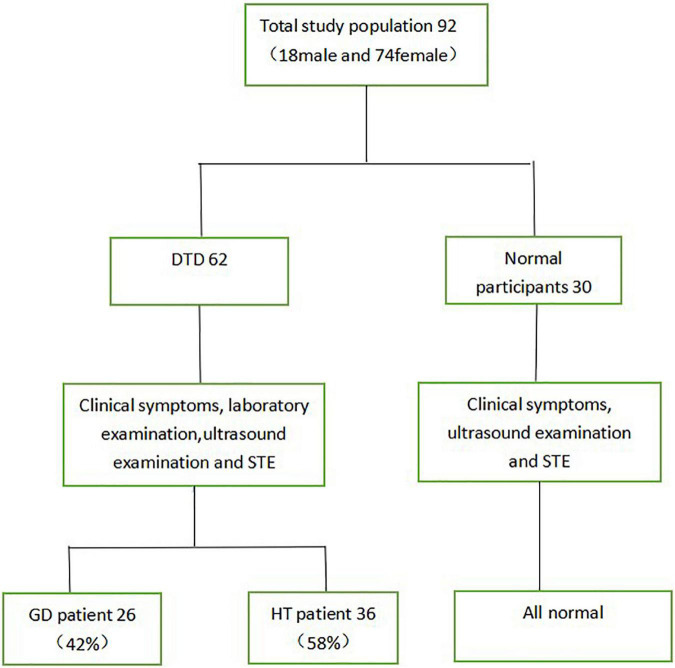
Flowchart of subject screening.

This study included 62 children with DTD seen at our hospital between March 2019 and March 2021, ranging in age from 3.9 to 17.2 years. The group comprised 6 boys and 56 girls, including 26 children with GD and 36 children with HT. Furthermore, 30 healthy controls, ranging in age from 5.2 to 14.3 years, with 12 boys and 18 girls (Figure 1).

Inclusion criteria for the GD group (19): (1) Clinical symptoms and signs of hyperthyroidism; (2) diffuse enlargement of the thyroid gland (3) elevated thyroid hormone concentrations and decreased serum TSH concentrations; (4) positive serum TRAb or TSAb can be used as an auxiliary diagnosis.

Inclusion criteria for the HT group (20): (1) Diffuse enlargement of the thyroid gland (2) positive serum TPOAb and TgAb; (3) increased serum TSH concentration can be used as an auxiliary diagnosis.

Exclusion criteria: (1) thyroid gland previously treated with surgery, radiation, drugs, etc.; (2) those with obvious thyroid nodules; (3) autoimmune diseases of other organs of the thyroid gland; (4) those with multiple combined diseases; (5) those with critical clinical conditions.

According to the above criteria, we excluded 4 children who could not be diagnosed with GD or HT by clinical symptoms, blood tests and ultrasound, 1 child with other immune system diseases and 2 children with subacute thyroiditis, and children who did not encounter goiter but had normal blood tests. We enrolled 92 children with DTD into the study.

Normal control group was comprised of 30 healthy volunteers. These subjects showed no abnormalities in thyroid gray-scale and Doppler ultrasound, no clinical symptoms and no history of thyroid disease.

### Measurement of conventional ultrasound and sound touch elastography

All ultrasound examinations were carried out by a single expert, who had 10 years of ultrasound experience and 5 years of UE experience, and used the same ultrasound system (Mindray Resona7, China) with a 3–11 MHz linear array probe for conventional ultrasound and STE. The thyroid gland was examined with conventional ultrasound, and the anterior and posterior diameters of the both lobes of the thyroid gland and the isthmus, bilateral lobe flow, and bilateral superior thyroid artery flow velocity and resistance index were measured. Longitudinal sectioning was conducted to minimize the impact of the common carotid artery pulsation and tracheal architecture on the elastic measurements,. The following elasticity parameters were determined for this region: mean Young’s modulus (Mean), maximum Young’s modulus (Max), minimum Young’s modulus (Min), and standard deviation (SD). To achieve the average value, at least six (three on the left and three on the right) elasticity parameter maps were produced for each participant.

Ultrasound color vascular assessment grading system was created: Grade 0: indicates that there is minimal to no blood flow in the gland; Grade 1: Mild blood flow signal can be detected in the gland, with mostly peripheral blood vessels visualized within an area less than one-third of that of a normal thyroid gland; Grade 2 indicates minimal hyperemia, with a distribution area of 1/3–2/3 of the gland; Grade 3 represents significant hyperemia increase in blood the gland, with a distribution area of at least more than 2/3, indicating a “sea of fire” appearance.

### Thyroid function

Each DTD participant had thyroid function testing and serum antibody assays performed, including Thyroid-stimulating Hormone (TSH), Free Triiodothyronine (FT3), Free Thyroxine (FT4), Thyroperoxidase Antibody (TPOAb), and Thyroglobulin Antibody (TgAb).

### Statistical analysis

SPSS24.0 statistical software was used for analysis, and the measurement data were expressed as $\bar{X}$ ± S. Multiple independent samples were tested using the one-way ANOVA method, and two comparisons were made between groups; the LSD test was used for chi-square, and the Games-Howell test was used for chi-square; rank data were tested using the rank sum test, and two comparisons were made between multiple independent samples using the SNK method. The receiver operating characteristic curve (ROC curve) analysis was used to assess the diagnostic efficacy of STE-Mean values in children with DTD and normal controls, as well as the diagnostic efficacy of superior thyroid artery flow velocity in children with GD and HT; the correlation between STE-Mean values and Vmax values of superior thyroid artery flow velocity in each thyroid group and laboratory. Pearson’s product distance correlation analysis was performed to assess the relationship between the thyroid gland groups’ STE-Mean values and PSV and the laboratory parameters; *P* < 0.05 indicates that the differences were statistically significant.

## Results

### Participants

In this study, participants included 62 children with DTD and 30 healthy volunteers. Children with DTD included 6 boys and 56 girls. The mean ages of GD group, HT group and normal group were 9.73 ± 3.56 years (range: 6.7–17.2 years), 11.46 ± 2.94 years (range: 3.9–16.9 years) and 8.06 ± 2.74 years (range: 5.2–14.3 years), respectively (Table 1). Of the 62 children with DTD, 26 had GD, all with hyperthyroidism, of which 24 received medication following the present examination; 36 had HT, 6 with hyperthyroidism, 6 with hypothyroidism, and 24 with normal thyroid function, and 8 received medication following the current examination.

**TABLE 1:** Routine ultrasound and elastic results in studied groups.

	NG	GD	HT	P
Sex(boys/girls)	30 (12/18)	26 (4/22)	36 (2/34)	0.041
Age(years)	8.06 ± 2.74	9.73 ± 3.56	11.46 ± 2.94	0.011
APD of isthmus (mm)	1.85 ± 0.49	5.15 ± 1.81**	4.43 ± 1.76**	<0.001
APD of left (mm)	10.89 ± 2.26	18.01 ± 3.10**	17.04 ± 2.45**	<0.001
APD of right (mm)	10.87 ± 1.90	18.79 ± 3.57**	17.45 ± 2.67**	<0.001
Blood flow rating	Grade1 (30/30)#	Grade3 (26/26)#	Grade1 (4/36)# Grade2 (14/36) Grade3 (18/36)	<0.001
Left PSV (cm/s)	20.76 ± 5.00#	87.87 ± 28.11#	52.16 ± 21.49#	<0.001
Left RI	0.64 ± 0.04	0.65 ± 0.06	0.61 ± 0.07	0.087
Right PSV (cm/s)	21.42 ± 3.27#	87.96 ± 29.27#	50.62 ± 19.24#	<0.001
Right RI	0.63 ± 0.04	0.67 ± 0.06	0.62 ± 0.07	0.073
STE-Mean (kPa)	11.24 ± 1.99	19.35 ± 5.00**	19.43 ± 6.06**	<0.001
STE-Max (kPa)	36.36 ± 9.03	52.04 ± 14.04**	53.96 ± 17.74**	<0.001
STE-Min (kPa)	3.02 ± 1.72	7.35 ± 4.33**	6.36 ± 3.68**	<0.001
STE-SD	4.75 ± 1.05	6.23 ± 1.64*	6.49 ± 2.35*	<0.001

NG, normal group; GD, Graves’ disease group; HT, Hashimoto’s thyroiditis group; APD, anteroposterior diameter. *: vs. NG, *P* < 0.05; **: vs. NG, *P* < 0.01; #: Comparison between groups, *P* < 0.01.

### Gray scale ultrasound

Thyroid volume was variably increased in GD and HT groups, and the anterior-posterior diameter of the isthmus, anterior-posterior diameter of the left lobe, and anterior-posterior diameter of the right lobe were larger than in the normal group, *P* < 0.05 (Table 1). The differences between the GD and HT groups, however, were not statistically significant (*P* > 0.05).

### Color Doppler ultrasound

The thyroid blood flow signal in the control group was evaluated as grade 1 (27/27). In the GD group, flow was increased significantly with a “sea of fire” appearance and overall grade of 3 (26/26). In the HT group, the majority of patients demonstrated increased flow (grade 1 4/36, grade 2 14/36, and grade 3 18/36). *P* < 0.05 for the comparison between groups (Table 1). However, when the blood flow increased significantly (grade 3), it was difficult to distinguish GD from HT.

In the comparison of superior thyroid artery flow velocity, GD group was elevated compared to HT group which in turn was elevated compared to the control group, *P* < 0.05. However, when comparing the superior thyroid artery flow resistance index between the groups the difference was not statistically significant, *P* > 0.05 (Table 1).

### Sound touch elastography measurements

**FIGURE 2:**
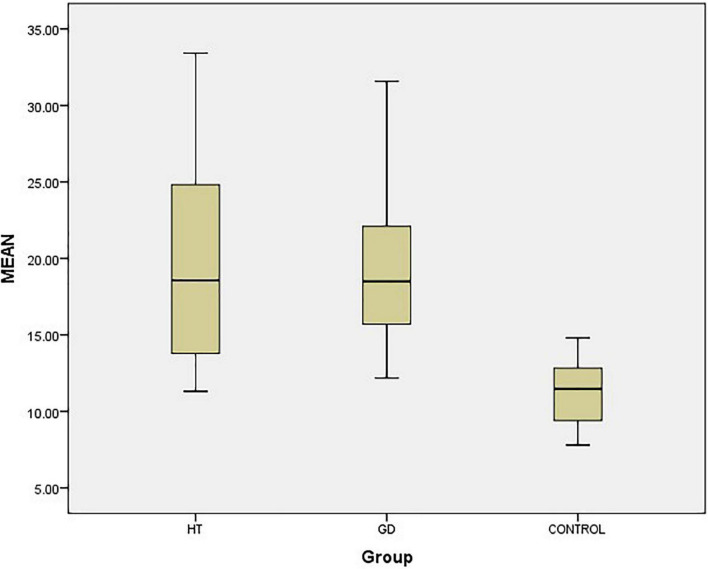
Box-plot of thyroid STE-Mean value. The top and bottom line segments represent the maximum and minimum values of data, respectively, wherein the top and bottom line segments of the box graph represent the third quartile and the first quartile, respectively, and the thick line segment in the middle of the box graph represents the median of data. CONTROL: normal group.

**FIGURE 3:**
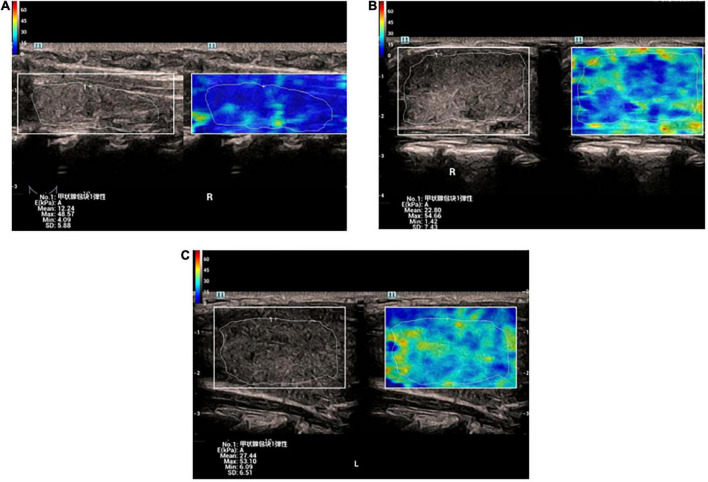
**(A)** Elastic image of normal thyroid: the sampling area is mainly blue, indicating that the gland is soft. The hardness increases gradually from blue-green-yellow-red. **(B)** Elastic image of Graves’ disease: the lesion area is mixed with blue and green. **(C)** Elastic image of Graves’ disease: the lesion area is mixed with blue and green.

The elastic parameters were selected as mean Young’s modulus (Mean), maximum Young’s modulus (Max), minimum Young’s modulus (Min), and SD, which were smaller in the normal group than in the lesion group in the four data groups, *P* < 0.05, while there was no statistical difference between the GD group and the HT group in all four data groups, *P* > 0.05 (Table 1 and Figures 2, 3A–C).

### Receiver operating characteristic curve analysis

**FIGURE 4:**
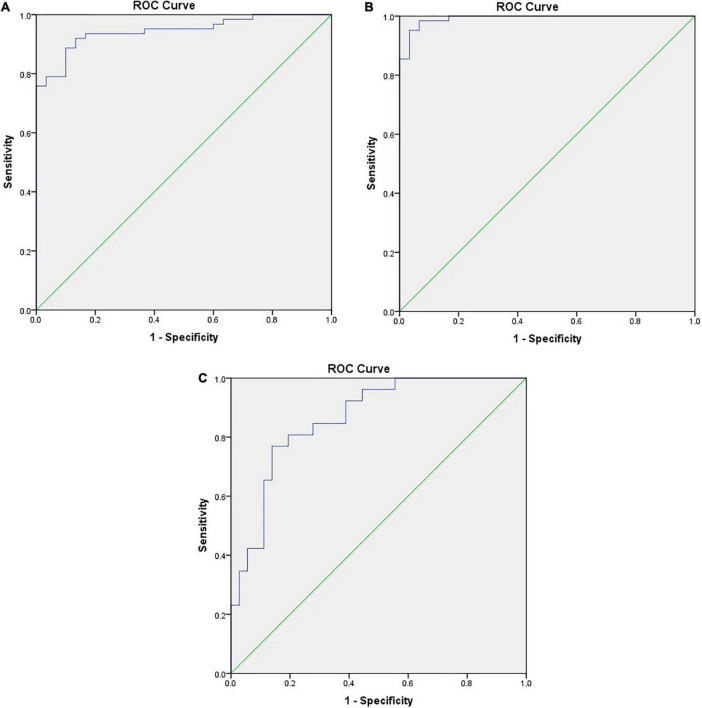
**(A)** ROC curve between DTD and NG differentiated by STE-Mean, the positive actual state is DTD. **(B)** ROC curve of DTD and NG differentiated by flow velocity of superior thyroid artery, the positive actual state is DTD. **(C)** ROC curve of GD and HT differentiated by flow velocity of superior thyroid artery, the positive actual state is GD.

ROC curve analysis was utilized to distinguish DTD children from normal children, and when the STE-Mean ideal cut-off value was 13.41 kPa, the diagnostic sensitivity was 91.9%, the specificity was 86.7%, and the AUC was 0.945 (Figure 4A).

When the PSV of superior thyroid artery was 25.39 cm/s, the diagnostic sensitivity was 98.4%, the specificity was 93.3%, and the AUC was 0.992 (Figure 4B). When the PSV of superior thyroid artery was 64.64 cm/s, the diagnostic sensitivity was 76.9%, the specificity was 86.1%, and the AUC was 0.864 (Figure 4C).

### Correlation analysis

In children with DTD, STE-Mean readings did not correlate substantially with FT3, FT4, TSH, TgAb, or TPOAb (Table 2). The PSV of superior thyroid artery exhibited a moderate positive association with FT3 and FT4, a low correlation with TgAb, and no significant correlation with TSH and TPOAb (Table 3).

**TABLE 2:** Correlation between STE-Mean value and serum indexes.

		FT3	FT4	TSH	TgAb	TPOAb
	r	0.181	0.117	0.168	0.128	0.208
**STE-Mean**						
	P	0.159	0.367	0.193	0.320	0.105

r: Correlation coefficient.

**TABLE 3:** Correlation between PSV of superior thyroid artery and serum indexes.

		FT3	FT4	TSH	TgAb	TPOAb
	r	0.602	0.593	–0.229	0.271	0.113
**PSV**						
	P	<0.001	<0.001	0.073	0.033	0.383

## Discussion

GD and HT are theorized to be secondary to an autoimmune response as an unknown antigen or various factors stimulating the production of antibodies such as TPOAb, TgAb, TRAb, etc. In addition, lymphocytes and plasma cells infiltrate the thyroid glands, accompanied by changes in blood vessels and proliferation of fibrous connective tissue (21). The normal thyroid is soft, with its basic structure is composed of follicles with a small amount of connective tissue, blood vessels and lymphatic vessels between the follicles (22). The pathological changes of GD are hyperplasia of follicular epithelial cells, scattered lymphocytes and plasma cells among the follicles, abundant and congested blood vessels in the stroma, and thin, reduced or disappeared glia in the follicles (22). The pathological changes of HT are extensive. In the early stage, there is a decrease of glia in the follicles, infiltration of lymphocytes and plasma cells between the follicles, proliferation and congestion of blood vessels in varying degrees with less proliferation of fibrous tissue. Gradually there is increase of lymphocyte infiltration. In its later stage there is severe atrophy and destruction of follicles, and proliferation of fibrous connective tissue in varying degrees, and even extensive fibrosis (22), which ultimately leads to the increase of gland hardness.

According to the findings of this study, STE-Mean, STE-Max, STE-Min, and STE-SD values were higher in the GD and HT groups than in the control group, with STE-Mean values of 19.35 ± 5.00, 19.43 ± 6.06, and 11.24 ± 1.99 kPa, respectively, indicating that the thyroid gland of children with DTD was stiffer than that of normal children. Diagnostic sensitivity was 91.9% and the specificity was 86.7%, with an AUC of 0.945 at the optimal STE-Mean cut-off value of 13.41 kPa, according to the findings of ROC curve analysis. The difference in stiffness between the GD and HT groups, however, was not statistically significant, indicating that STE is ineffective for differentiating between these disorders. Hazem et al. (23) used the SWE technique on 74 children with DTD and 20 normal children ranging in age from 10 to 18 years, and SWE-Mean values of 17.26 ± 4.2, 15.31 ± 2.95, and 10.9 ± 1.78 kPa were found in the GD, HT, and normal groups, respectively, Their study demonstrated statistically significant differences between the groups, with the GD group demonstrating increased stiffness compared to the HT group, which differed from the results of our study. Liu et al. (24) measured the SWE technique in adult 154 patients with diffuse thyroid lesions and 30 normal controls, ages 13–74 years, demonstrating SWE-Mean values of 27.05 ± 12.21, 26.11 ± 11.90, 13.88 ± 4.10 kPa in the GD, HT, and normal groups, respectively, similar to our current study of DTD. DTD demonstrated increased stiffness compared to the normal group, without statistical significance. The STE-Mean values of all groups were somewhat higher than those of the current research. This may be related to the duration of the disease, and the subtle gland fibrosis in children (20). In addition, Kandemirli et al. (25) assessed the degree of fibrosis in 59 children with HT and 27 normal children, aged 7.5–17.0 years, using SWE technology, and classified HT into three levels. The STE-mean value of children with grade 3 (19.7 kpa, IQR17.9–21.5 kpa), which was higher than that of children with grade 2 (15.5 kpa, IQR14.5–17.8 kpa) and higher than that of children with grade 1 (12.8 kpa, IQR11.9–13.1 kpa) and higher than that of normal children (10.6 kpa, IQR9.0–11.3 kpa). The difference between the two groups was statistically significant, indicating that the glandular fibrosis of HT was different in different stages of disease development.

The SWE technique to assess tissue hardness can be expressed in kPa in addition to the shear wave velocity (SWV) unit cm/s, with a relationship between the two expressed by the formula E = 3 pc2 (E = Young’s modulus, p = tissue density, c = SWV). However, the differences in the techniques of different manufacturers, which assess the stiffness in different units, cannot be converted artificially. Yucel et al. (26) examined the SWV of the thyroid gland in 26 children with HT and 26 normal children, ages 6–17 years, and discovered that the SWV value was higher in the HT group (1.67 ± 0.63 cm/s) than in the normal group (1.30 ± 0.13 cm/s, *p* < 0.001), indicating that the thyroid stiffness of HT group is harder than that of normal group, which is consistent with the current study. Another thyroid investigation in 145 healthy children found normal thyroid SWV value of 1.22 ± 0.20 cm/s and no association between SWV and age, BSA, or thyroid volume (27). In addition, Hefeda (28) compared the thyroid gland of 130 adults, including 30 GD patients, 65 HT patients and 35 normal people, aged 18–52 years. The SWV values of GD group, HT group and control group were 2.61 ± 0.32, 2.85 ± 0.52 and 1.75 ± 0.37 m/s, respectively. There were statistical differences among the groups. The HT group was slightly harder than GD group, and both were harder than the control group. Their data was slightly different from our study, possibly because DTD lasts longer in adults, and gland fibrosis is more severe.

In our study population, children with GD and HT, routine ultrasound findings revealed identical gray scale sonograms with varied degrees of glandular expansion and heterogeneous echogenicity. Color Doppler showed abundant blood flow in GD, and blood flow were all grade 3, with obvious vascular pulsation, which was related to the obvious increase of blood vessels and the rapid flow rate (29). While in HT patients, most of the blood flow is slightly increased or increased, mainly concentrated in grade 2 and grade 3. This may be related to inflammation and increased vascular growth factor (30). Some literatures (30, 31) has suggested early that the blood supply pattern of some HT and Gd was similarly increased. Ceylan et al. (32) also reported that 85% of HT patients had increased vascularity. Although the two overlapped, the flow rate was not as fast as that of GD patients, which was also similar to the results of previous literatures (5–7). ROC curve analysis revealed that the optimal cut-off value of the PSV of superior thyroid artery was 64.64 cm/s, yielding a diagnostic sensitivity of 76.9%, and specificity of 86.1%, and AUC was 0.864. Caruso et al. (7) reported that the peak systolic velocity (PSV) of the inferior thyroid artery in GD patients was > 150 cm/s, and the PSV of other autoimmune thyroiditis patients was < 65 cm/s. We also discovered that the PSV of the superior thyroid artery correlated moderately with FT3 and FT4 (FT3:Pearson *r* = 0.602, *P* < 0.001; FT4:Pearson *r* = 0.593; *P* < 0.001), and not significantly linked with TSH, TgAb or TPOAb, suggesting that, while higher blood flow does not predict hyperthyroidism, faster flow rate may be indicative of hyperthyroidism.

Whereas in previous literature demonstration of the link between serum markers and DTD stiffness varied substantially, Yucel et al. (26) revealed that thyroid SWV in HT patients associated with TPOAb (Pearson *r* = 0.46; *P* = 0.038) but not with FT3, FT4, TSH, or TgAb. Thyroid SWE in DTD patients revealed a modest association with TSH and TgAb (*r* = 0.269, *P* = 0.025; *r* = 0.343, *P* = 0.004) and no significant correlation with FT3, FT4, and TPOAb, according to Liu et al. (24). In our study STE-Mean levels were not significantly linked with FT3, FT4, TSH, TgAb, or TPOAb in children with DTD. Therefore, we are not yet capable of predicting thyroid function based on hardness.

The limitations of this study include the limited sample size, and short follow up, which did not allow for the observation of changes in stiffness as the disease progressed; second, the use of UE in superficial organs is subject to interference from a variety of causes.

## Conclusion

In conclusion, STE of the thyroid can aid in differentiating between normal children and children with DTD, however, STE cannot reliably differentiate between GD and HT, and the blood flow velocity of the superior thyroid artery can be a good supplement to the differential diagnosis of the both. Hopefully with added research and larger sample sizes the role of UE in children with DTD can be verified.

## Data availability statement

The original contributions presented in the study are included in the article/supplementary material, further inquiries can be directed to the corresponding author/s.

## Ethics statement

The studies involving human participants were reviewed and approved by the Medical Research Ethics Review Committee of Suining Central Hospital. Written informed consent to participate in this study was provided by the participants’ legal guardian/next of kin.

## Author contributions

LL: subject design, data collection, statistical analysis, and article writing. AZ: data collection and statistical analysis. DC: data collection. BT: subject guidance and article modification. XL: subject guidance and quality control. All authors contributed to the article and approved the submitted version.

## References

[1] Okayasu I Hara Y Nakamura K Rose NR. Racial and age-related differences in incidence and severity of focal autoimmune thyroiditis. *Am J Clin Pathol.* (1994) 101:698–702. 10.1093/ajcp/101.6.698 8209854

[2] Antonelli A Ferrari SM Corrado A Di Domenicantonio A Fallahi P. Autoimmune thyroid disorders. *Autoimmun Rev.* (2015) 14:174–80. 10.1016/j.autrev.2014.10.016 25461470

[3] Pishdad P Pishdad GR Tavanaa S Pishdad R Jalli R. Thyroid ultrasonography in differentiation between graves’ disease and hashimoto’s thyroiditis. *J Biomed Phys Eng.* (2017) 7:21–6. 28451576 PMC5401130

[4] Corona G Biagini C Rotondi M Bonamano A Cremonini N Petrone L Correlation between clinical, biochemical, color Doppler ultrasound thyroid parameters, and CXCL-10 in autoimmune thyroid diseases. *Endocr J.* (2008) 55:345–50. 10.1507/endocrj.k07e-052 18379127

[5] Donkol RH Nada AM Boughattas S. Role of color Doppler in differentiation of Graves’ disease and thyroiditis in thyrotoxicosis. *World J Radiol.* (2013) 5:178. 10.4329/wjr.v5.i4.178 23671754 PMC3647210

[6] Schraml C Müssig K Martirosian P Schwenzer NF Claussen CD Häring HU Autoimmune thyroid disease: arterial spin-labeling perfusion MR imaging. *Radiology.* (2009) 253:435–42. 10.1148/radiol.2533090166 19789231

[7] Caruso G Attard M Caronia A Lagalla R. Color Doppler measurement of blood flow in the inferior thyroid artery in patients with autoimmune thyroid diseases. *Eur J Radiol.* (2000) 36:5–10. 10.1016/S0720-048X(00)00147-9 10996751

[8] Thumar V Squires JH Spicer PJ Robinson AL Chan SS. Ultrasound elastography applications in pediatrics. *Ultrasound Q.* (2018) 34:199–205. 10.1097/RUQ.0000000000000379 30169493

[9] Højte C Jørgensen MH Jensen F Katzenstein TL Skov M. Extended screening for cystic fibrosis-related liver disease including elastography in children and adolescents. *J Pediatr Gastroenterol Nutr.* (2020) 71:663–8. 10.1097/MPG.0000000000002872 33093375

[10] Ferraioli G Barr RG Dillman JR. Elastography for pediatric chronic liver disease: a review and expert opinion. *J Ultrasound Med.* (2021) 40:909–28. 32881048 10.1002/jum.15482

[11] Dietrich CF Ferraioli G Sirli R Popescu A Sporea I Pienar C General advice in ultrasound based elastography of pediatric patients. *Med Ultrasonogr.* (2019) 21:315–26. 10.11152/mu-2063 31476212

[12] Desvignes C Dabadie A Aschero A Ruocco A Garaix F Daniel L Technical feasibility and correlations between shear-wave elastography and histology in kidney fibrosis in children. *Pediatr Radiol.* (2021) 51:1879–88. 10.1007/s00247-021-05068-x 33893848

[13] Elgendy A Elhawary E Shareef MM Romeih M Ebeed A. Ultrasound elastography in the diagnosis of malignant cervical lymphadenopathy in children: can it replace surgical biopsy? *Eur J Pediatr Surg.* (2022) 32:321–326. 10.1055/s-0041-1729900 34091882

[14] Hanquinet S Courvoisier D Kanavaki A Dhouib A Anooshiravani M. Acoustic radiation force impulse imaging—normal values of liver stiffness in healthy children. *Pediatr Radiol.* (2013) 43:539–44. 10.1007/s00247-012-2553-5 23247632

[15] Riquier-Le Chatelier M Giai J Lallement-Dudek P Herisson O Fitoussi F. Muscle elasticity in patients with neonatal brachial plexus palsy using shear-wave ultrasound elastography. Preliminary results. *J Pediatr Orthopaed B.* (2021) 30:385–92. 10.1097/BPB.0000000000000781 34031322

[16] Kim HG Park MS Lee JD Park SY. Ultrasound elastography of the neonatal brain: preliminary study. *J Ultrasound Med.* (2017) 36:1313–9. 10.7863/ultra.16.06079 28304105

[17] Saǧlam D Demirbaş F Bilgici MC Yücel S Çaltepe G Eren E. Can point shear wave elastography be used as an early indicator of involvement? Evaluation of the pancreas and liver in children with cystic fibrosis. *J Ultrasound Med.* (2020) 39:1769–76. 10.1002/jum.15281 32309883

[18] Sǎftoiu A Gilja OH Sidhu PS Dietrich CF Cantisani V Amy D The EFSUMB guidelines and recommendations for the clinical practice of elastography in non-hepatic applications: update 2018. *Ultraschall Med Eur J Ultrasound.* (2019) 40:425–53. 10.1055/a-0838-9937 31238377

[19] Endocrinology Branch of Chinese Medical Association. Chinese guidelines for diagnosis and treatment of thyroid disease. *Chin J Int Med.* (2007) 46:876–82.

[20] Caturegli P De Remigis A Rose NR. Hashimoto thyroiditis: clinical and diagnostic criteria. *Autoimmun Rev.* (2014) 13:391–7. 10.1016/j.autrev.2014.01.007 24434360

[21] Lee HJ Li CW Hammerstad SS Stefan M Tomer Y. Immunogenetics of autoimmune thyroid diseases: a comprehensive review. *J Autoimmun.* (2015) 64:82–90. 10.1016/j.jaut.2015.07.009 26235382 PMC4628844

[22] Goldblum JR Lamps LW McKenney J Myers JL. *Rosai and Ackerman’s Surgical Pathology.* Philadelphia, PA: Elsevier (2018). p. 278–90.

[23] Hazem M Khalid Al Jabr I AlYahya AA Hassanein AG Elghany Algahlan HA. Reliability of shear wave elastography in the evaluation of diffuse thyroid diseases in children and adolescents. *Eur J Radiol.* (2021) 143:109942. 10.1016/j.ejrad.2021.109942 34479126

[24] Liu J Zhang Y Ji Y Wan Q Dun G. The value of shear wave elastography in diffuse thyroid disease. *Clin Imaging.* (2018) 49:187–92. 10.1016/j.clinimag.2018.03.019 29627743

[25] Kandemirli SG Bayramoglu Z Caliskan E Sari ZNA Adaletli I. Quantitative assessment of thyroid gland elasticity with shear-wave elastography in pediatric patients with Hashimoto’s thyroiditis. *J Med Ultrason.* (2018) 45:417–23. 10.1007/s10396-018-0859-0 29349581

[26] Yucel S Ceyhan Bilgici M Kara C Can Yilmaz G Aydin HM Elmali M Acoustic radiation force impulse quantification in the evaluation of thyroid elasticity in pediatric patients with Hashimoto thyroiditis. *J Ultrasound Med.* (2018) 37:1143–9. 10.1002/jum.14459 29064111

[27] Ceyhan Bilgici M Saðlam D Delibalta S Yücel S Tomak L Elmal M. Shear wave velocity of the healthy thyroid gland in children with acoustic radiation force impulse elastography. *J Med Ultrason.* (2018) 45:75–80. 10.1007/s10396-017-0788-3 28424923

[28] Hefeda MM. Value of the new elastography technique using acoustic radiation force impulse in differentiation between Hashimoto’s thyroiditis and Graves’ disease. *J Clin Imaging Sci.* (2019) 9:17. 10.25259/JCIS-22-2019 31448168 PMC6702860

[29] Ralls PW Mayekawa DS Lee KP Colletti PM Radin DR Boswell WD Color-flow Doppler sonography in Graves disease: “thyroid inferno”. *Am J Roentgenol.* (1988) 150:781–4. 10.2214/ajr.150.4.781 3279732

[30] Iitaka M Miura S Yamanaka K Kawasaki S Kitahama S Kawakami Y Increased serum vascular endothelial growth factor levels and intrathyroidal vascular area in patients with graves’ disease and Hashimoto’s thyroiditis. *J Clin Endocrinol Metab.* (1998) 83:3908–12. 10.1210/jcem.83.11.5281 9814467

[31] Vitti P Rago T Mazzeo S Brogioni S Lampis M De Liperi A Thyroid blood flow evaluation by color-flow Doppler sonography distinguishes graves’ disease from Hashimoto’s thyroiditis. *J Endocrinol Investig.* (1995) 18:857–61. 10.1007/BF03349833 8778158

[32] Ceylan I Yener S Bayraktar F Secil M. Roles of ultrasound and power Doppler ultrasound for diagnosis of hashimoto thyroiditis in anti-thyroid marker-positive euthyroid subject. *Quant Imaging Med Surg.* (2014) 4:232–8. 10.3978/j.issn.2223-4292.2014.07.13 25202658 PMC4137176

